# Supplementary material for: Indigeneity and Likelihood of Discharge to Psychiatric Hospital in an Australian Deliberate Self-Poisoning Hospital-Treated Cohort
Source: Int J Environ Res Public Health. 2022 Sep 27;19(19):12238. doi: 10.3390/ijerph191912238 (PMC9566708; doi:10.3390/ijerph191912238)
Supplement: Supplementary file 1 [file ijerph-19-12238-s001.zip › ijerph-1895002-SI.pdf]

Table S1. Univariate Logistic Regressions for Discharge to Psychiatric Hospital.

Total Cohort (n=3846)	*n* discharged to psychiatric hospital	*n* total	OR	95% CI	p value
**Age Group**					
18–25 years	248	1000	1.0		
26–40 years	442	1415	1.38	(1.15–1.65)	.001
41–50 years	275	792	1.61	(1.31–1.98)	<.001
51–60 years	162	413	1.96	(1.53–2.50)	<.001
61+ years	80	226	1.66	(1.22–2.26)	.001
**Gender**					
Female	689	2389	1.0		
Male	518	1457	1.36	(1.18–1.56)	<.001
**Employment**					
Employed	301	1105	1.0		
Not In paid work	515	1495	1.40	(1.18–1.66)	<.001
Unknown	391	1246	1.22	(1.02–1.46)	.028
**Marital Status**					
Married or de facto	375	1219	1.0		
Single/never married	584	1949	0.96	(0.82–1.13)	.634
Divorced, widowed or separated	229	638	1.26	(1.03–1.54)	.025
**Housing**					
Housing	1066	3534	1.0		
Homeless	56	110	2.40	(1.64–3.51)	<.001
**Highest Education**					
Primary or Secondary	872	2903	1.0		

Total Cohort (n=3846)	*n* discharged to psychiatric hospital	*n* total	OR	95% CI	p value
Tertiary	184	596	1.04	(0.86–1.26)	.686
**Suicidal level at assessment**					
No thoughts or plan	210	2394	1.0		
Low to moderate suicidal ideation and no plan	365	674	12.29	(9.98–15.12)	<.001
Plan or strong/intense/pervasive suicidal ideation	632	778	45.02	(35.81–56.61)	<.001
**Psychiatric Contact in past 12 months**					
None	345	1474	1.0		
Outpatient contact (only)	512	1691	1.42	(1.21–1.67)	<.001
Inpatient contact (any)	350	681	3.46	(2.86–4.20)	<.001
**Anxiety Disorder**					
No	1061	3373	1.0		
Yes	146	473	0.97	(0.79–1.20)	.796
**Mood Disorder**					
No	446	2043	1.0		
Yes	761	1803	2.62	(2.27–3.01)	<.001
**Relationship Problem (V code)**					
No	725	1932	1.0		
Yes	482	1914	0.56	(0.49–0.64)	<.001
**Personality Disorder**					
No	966	3126	1.0		
Yes	241	720	1.13	(0.95–1.34)	.180
**Substance Use Disorder**					
No	637	1944	1.0		
Yes	570	1902	0.88	(0.77–1.01)	.062

Total Cohort (n=3846)	*n* discharged to psychiatric hospital	*n* total	OR	95% CI	p value
**Schizophrenia & Other Psychotic Disorder**					
No	1058	3603	1.0		
Yes	149	243	3.81	(2.91–4.99)	<.001
**Other Diagnosis**					
No	989	3039	1.0		
Yes	218	807	0 .77	(0.65–0.91)	.003
**Alcohol co-ingested**					
No alcohol	800	2349	1.0		
Alcohol co-ingested	407	1497	0.72	(0.63–0.83)	<.001
